# Turnover intention of foreign trained physicians in German rehabilitation facilities—a quantitative study

**DOI:** 10.1186/s12913-024-10902-7

**Published:** 2024-03-29

**Authors:** Eva Jansen, Johanna Schmidt, Manuela Marquardt

**Affiliations:** Charité—Universitätsmedizin Berlin, Berlin, Germany

**Keywords:** Retention, Turnover intention, Foreign trained Physicians, Rehabilitation, Health Workforce, International Medical graduates, Migrated Physicians

## Abstract

**Background:**

Germany’s medical specialist shortage is an acute challenge, especially in the rehabilitation segment. One countermeasure is to recruit foreign trained physicians (FTP), but the high turnover of FTP is a burden on the departments that train them and integrate them professionally. Preliminary research showed that currently one in three physician positions in German Pension Insurance (DRV) contract facilities is filled by FTP.This paper examines factors related to turnover intention of FTP in German rehabilitative departments.

**Methodology:**

In spring 2022, we surveyed FTP across all inpatient and outpatient rehabilitation departments under the German Pension Insurance, using a two-stage cross-sectional approach. We conducted an online survey of FTP and developed a specialized questionnaire that captured sociodemographic, occupation related and professional biographical data, turnover intention, satisfaction, difficulties with professional integration and departmental structural characteristics. To analyze retention within the rehabilitation field, we used a measure of turnover intention, taking into account the direction of potential turnover, residency requirements and considerations of returning to the rehabilitation field. The data was evaluated in a subgroup analysis comparing FTP with and without turnover intention using Fisher’s exact tests.

**Results:**

The sample includes *n* = 145 FTP, 119 stating no turnover intention and 27 with turnover intention. More than half of FTP with turnover intention wished to move to an acute care hospital. FTP with turnover intention are comparatively younger and came to Germany and were employed in the rehabilitation departments more recently, indicating an earlier career stage. Besides, career-related and regional factors show the strongest relation to turnover intention.

**Discussion and conclusion:**

The results reveal a group of “established FTP” whose professional integration has been successfully completed. FTP with turnover intention are comparatively younger, career-oriented physicians for whom work in a rehabilitative facility is a career springboard to gain a foothold in acute care clinics. A limitation is that FTP with turnover intention are difficult to reach and may be underrepresented in our sample.

**Supplementary Information:**

The online version contains supplementary material available at 10.1186/s12913-024-10902-7.

## Background

A structural shortage of physicians is affecting not only low-income countries but also certain specialties and regions in high-income countries [1]. In Germany, this mainly affects general medicine and the public health service, as well as certain areas of rehabilitative medicine [2–4]. The former areas will experience an even greater future risk of shortages as currently practicing medical professionals retire [5]. Rehabilitative medicine will also face increased demand due to the rise in the aging population that will require treatment for age-related illnesses [6].

Rehabilitation plays a specific role in the German healthcare system: thetreatments are funded by the public pension insurance and aim to help patients recover and return to the labo r force (ICF-term of functional health) [7] as well as to support older people who are no longer working to maintain independence. The majority of rehabilitation in Germany occurs in inpatient settings (83%) [8]. To achieve treatment goals, physicians must understand both a patient’s medical profile and the psychosocial factors affecting their condition [9]. However, there is a shortage of physicians in this field, particularly among younger doctors who are less likely to join rehabilitation centers or engage in “social medicine” in general [10]. Additionally, rehabilitation centers are disproportionately frequently located in remote, economically disadvantaged areas [11], making it difficult to recruit young physicians from within Germany [12]. One strategy to address the shortage of physicians is to recruit foreign-trained physicians (FTP) from abroad. However, the professional integration of FTP is often associated with an additional burden for the receiving team and for the physicians themselves, especially in the initial phase. Previous studies on this topic indicate that FTP often have insufficient language and communication skills, particularly for the doctor-patient relationship [13, 14], but also for inter-professional communication [15]. Furthermore, FTP are unfamiliar with and lack knowledge about the national healthcare system, disease and therapy concepts, and patient-oriented, participatory approaches, all of which pose challenges in professional integration [16–18]. A specific characteristic of the practice of rehabilitation in Germany that is unusual for FTP are the forms of inter-hierarchical and inter-professional collaboration [19]. A qualitative preliminary study has shown that in rehabilitation, new tasks such as the expert function of physicians and the bureaucratic processes are the greatest challenges [20] and that for FTP social integration has significant relevance [21].

The strategy of filling staff gaps with FTP also comes with added instability to healthcare delivery, namely high physician turnover and subsequent discontinuity of patient care. The retention of healthcare workers is a crucial international workforce issue, yet the concept itself remains ambiguous [29]. As human resource theorists state, the concept of voluntary turnover should be elucidated as a combination of social, economic and psychological processes and not each individually [22, 23]. Turnover as a metric refers to people leaving a health institution either voluntarily or because of an employer’s decision, while retention refers to the rate at which people stay with a company over a period of time and the strategies employed to keep them. Although turnover intention does not refer to the act of leaving itself but rather describes the likelihood the physician will choose to do so, there is evidence that proves that actual turnover positively correlates with turnover intention [24, 25].

For all institutions, high levels of turnover create a loss of financial and social capital [26] and affect the morale of the remaining workforce as well as the reputation of the organization [27]. Turnover in any form is a drain on healthcare organization funding as it is expensive and time-consuming to recruit new health care workers into the organizational workforce. It is therefore valuable to consider how turnover can be reduced and retention increased [28].

In this study, we refer to partial results of the quantitative questionnaire study “Medical professionals with a foreign degree in German rehabilitation facilities—a quantitative cross-sectional study” in order to examine factors related to the retention of FTP within the field of rehabilitation.

## Methods

**Fig. 1:**
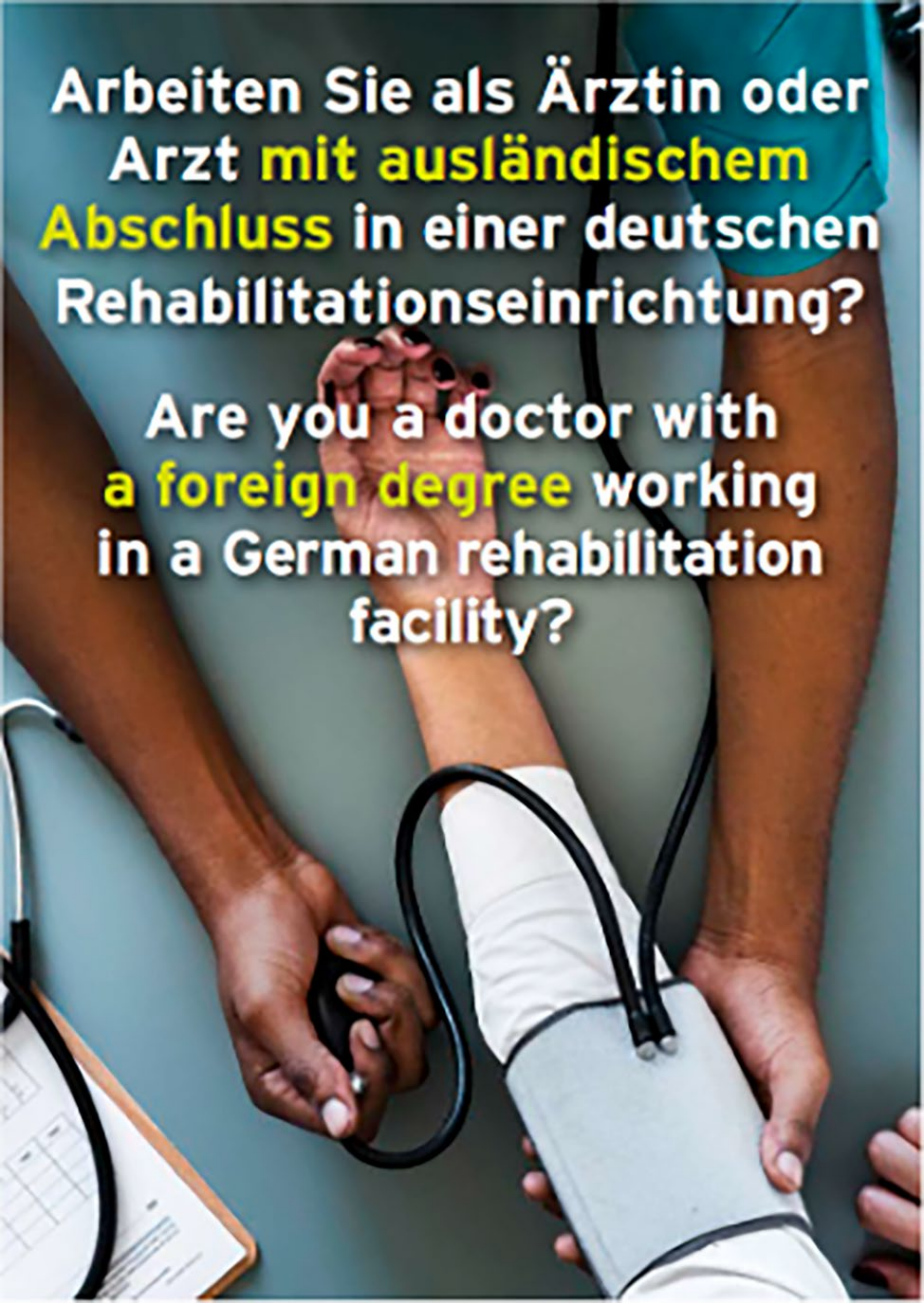
Flyer for the recruitment of FTP, front page

In spring 2022 we conducted a full survey in all inpatient and outpatient rehabilitation departments administered by the German Pension Insurance (DRV). We collected cross-sectional data in two stages: For one stage of the full survey, we wrote to the heads of the departments by mail and asked them to participate in the department survey. For the second stage we asked them to invite all FTP working for them to participate in the online FTP survey by means of the enclosed flyers (Fig. 1), that represents the database for the present analysis on retention. For this study, we defined FTP based on where they completed their studies (not in Germany). This does not include physicians who were born abroad, started studying abroad and changed their place of study during their studies, or who would otherwise fall into the category “with a migration background” in an otherwise undefined way (for different terminologies, see [29]).

As common definitions and standardized questionnaires are still lacking in the field of physician retention, we developed the FTP questionnaire tailored to the problem of our study—retention within the field of rehabilitation (supplement 1). We chose the relevant dimensions based on a qualitative study in which we conducted interviews with FTP in rehabilitation facilities [20] and other questionnaires that had already been carried out on the subject of physician retention [29].

The FTP survey included sociodemographic, professional biography and departmental structural characteristics as well as several items on the turnover intention. Furthermore, variables representing various aspects of work-related and general satisfaction with life in Germany as well as difficulties with professional integration and discrimination were included. To implement the online survey, we used the Evasys platform [30]. The survey was preceded by two pretests: one involving social science experts and another with the target audience. The questions were tested multiple times for clarity and accessibility. Additionally, an English version was available, allowing participants to switch if preferred.

For the subgroup comparison, a variable on retention in the field of rehabilitation was created using information on turnover intention, turnover direction and in case of intended changes due to residency requirements, whether going back to the rehabilitation field is an option.

To examine the differences in the distributions of sociodemographic, occupation and professional biography related, departmental structural variables between FTP with and without turnover intention, a subgroup analysis of the available cases was conducted using Fisher’s exact tests. Furthermore, variables on satisfaction and difficulties are compared using Likert plots. For dichotomous variables, both one-tailed and two-tailed p-values are reported. For variables with more than two levels, the Freeman-Halton extension of the Fisher exact test is used, reporting two-tailed significance. Due to the small group size of the FTP with turnover intention, the significant differences are interpreted as empirical tendencies.

## Results

**Fig. 2:**
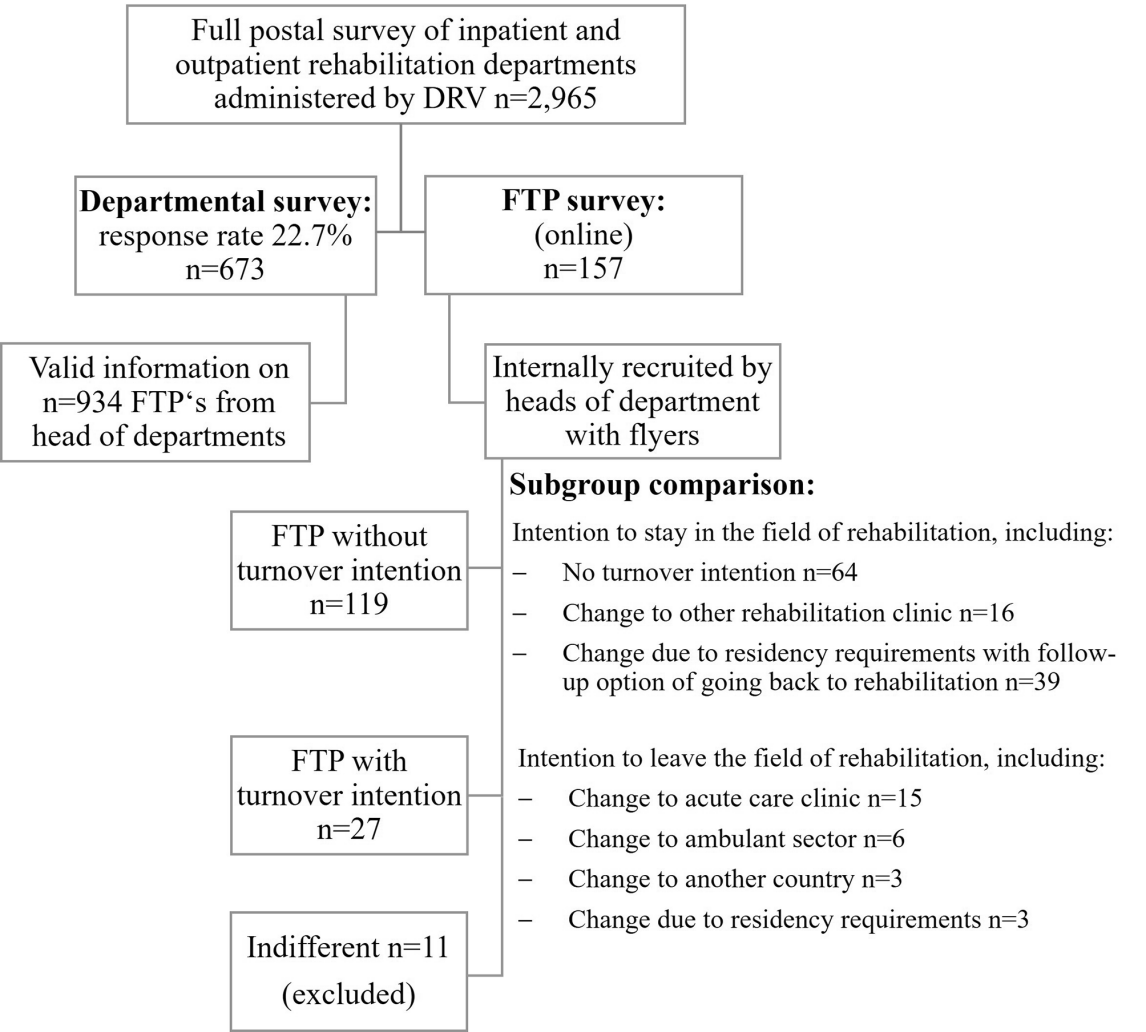
Flow chart of departmental and FTP survey with subgroup characterization

Figure 2 depicts the flow of participants in the departmental (results reported in another publication: 37) and the FTP survey. The group of FTP with turnover intention comprised *n* = 27 cases and the group without turnover intention *n* = 119.

Of the *n* = 27 FTP in the group with turnover intention, the most likely career change for 15 respondents (55.6%) is to change to the acute care sector in Germany, 6 (22.2%) want to go to the outpatient sector in Germany, and 3 (11.1%) to another country. Other 3 (11.1%) FTP intend to change jobs due to their residency requirements, but subsequently do not want to work in the field of rehabilitation.

The comparison of sociodemographic characteristics reveals that, in our sample, FTP with turnover intention are more likely to be male, younger, and less likely to have German citizenship compared to FTP without turnover intention. In both groups, living together with a partner and/or children in Germany is the predominant family situation. Occupation-related, they work more often on temporary contracts (40.7% - compared to 25.2% FTP without turnover intention) and with a provisional professional license^1^ (25.9% - compared to 14.3% without turnover intention). FTP without turnover intention have a German specialist title more often (34.5% - compared to 18.5% with turnover intention) and 15.1% occupy positions with leadership roles, compared to 0.0% in the FTP group with turnover intention. These characteristics suggest that FTP with turnover intentions are at an earlier stage in their professional careers. This is also reflected in the professional biographical variables—4 out of 10 have been in Germany for less than two years, and nearly half have been working in the rehabilitation department for less than a year. In the group without turnover intentions, two-thirds have been in Germany for more than four years, and 77.1% have been working in the department for over a year, with 62.7% of them for more than two years. Looking at the departmental structural characteristics, department size in terms of treatment places seems to be almost equally distributed between the subgroups with and without turnover intention. One structural distinction between the groups can be found in the FTP share among medical staff, with 7 out of 10 FTP with turnover intention working in departments where more than half of the medical staff was educated abroad compared to half of the FTP without turnover intention. The most significant subgroup difference is evident in the regional location of the employer’s facilities—57.1% of FTP with turnover intention work in departments located in peripheral areas and none in central urban areas, compared to 36.1% of FTP without turnover intention, with as many as 15.1% working in central city locations. These data are listed in Table 1, sorted by socio-demographic characteristics, occupational and occupational biographical characteristics and departmental structural characteristics.

**Table 1:** Subgroup comparison of FTP with and without turnover intention on sociodemographic, occupation-related and professional biographical, and departmental structural characteristics

	FTP with turnover intention		FTP without turnover intention		Total		Fisher’s exact test	
	n	%	n	%	n	%	1-tailed	2-tailed
Sociodemographic characteristics								
**Sex**	27		118		145		0.069	0,134
Male	17	63.0	53	44.9	70	48.3		
Female	10	37.0	65	55.1	75	51.7		
**Age**	27		119		146		< 0.001	< 0.001
26–35 years	18	66.6	36	30.3	54	37.0		
> 35 years	9	33.3	83	69.7	92	62.9		
**Nationality**	24		114		138		0.037	0.059
German	4	16.7	43	37.7	47	34.1		
Other	20	83.3	71	62.3	91	65.9		
Occupation-related and professional biographical characteristics								
**Temporary contract**	27		119		146		0.086	0.153
Yes	11	40.7	30	25.2	41	28.1		
No	16	59.3	89	74.8	105	71.9		
**Provisional professional license**	27		119		146		0.120	0.155
Yes	7	25.9	17	14.3	24	16.4		
No	20	74.1	102	85.7	122	83.6		
**German specialist title**	27		119		146		0.081	0.167
Yes	5	18.5	41	34.5	46	31.5		
No	22	81.5	78	65.5	100	68.5		
**Leading role**	27		119		146		0.019	0.025
Yes	0	0.0	18	15.1	18	12.3		
No	27	100.0	101	84.9	128	87.7		
**In Germany since**	27		119		146		-	0.001
≤ 1 year	3	11.1	4	3.4	7	4.8		
> 1–2 years	8	29.6	9	7.6	17	11.6		
> 2–4 years	6	22.2	24	20.2	30	20.5		
> 4 years	10	37.0	82	68.9	92	63.0		
**In department since**	27		118		145		-	< 0.001
≤ 1 year	12	44.4	27	22.9	39	26.9		
> 1–2 years	8	29.6	17	14.4	25	17.2		
> 2–4 years	6	22.2	25	21.2	31	21.4		
> 4 years	1	3.7	49	41.5	50	34.5		
Departmental structural characteristics								
**Department size**	22		114		136		-	0.676
1–49 treatment places	7	31.8	29	25.4	36	26.5		
50–99 treatment places	3	13.6	25	21.9	28	20.6		
100–500 treatment places	12	54.5	60	52.6	72	52.9		
**> 50% FTP share among medical staff**	26		115		141		0.046	0.081
Yes	18	69.2	56	48.7	74	52.5		
No	8	30.8	59	51.3	67	47.5		
BIK^a^	26		119		145		-	0.025
Periphery	15	57.7	43	36.1	58	40.0		
Environs	11	42.3	58	48.7	69	47.6		
Centers	0	0.0	18	15.1	18	12.4		

^a^The BIK region size classes are a nationwide typology of settlement structure that takes into account commuting priorities, population/job density, and population size of the entire region when defining linkage areas. For this analysis, the typology was condensed to three categories: Periphery (BIK-regions up to 50,000 inhabitants), Environs (Surrounding municipalities of BIK-regions with 50,000 or more inhabitants) and Centers (Core areas of BIK-regions with 50,000 or more inhabitants).

**Fig. 3:**
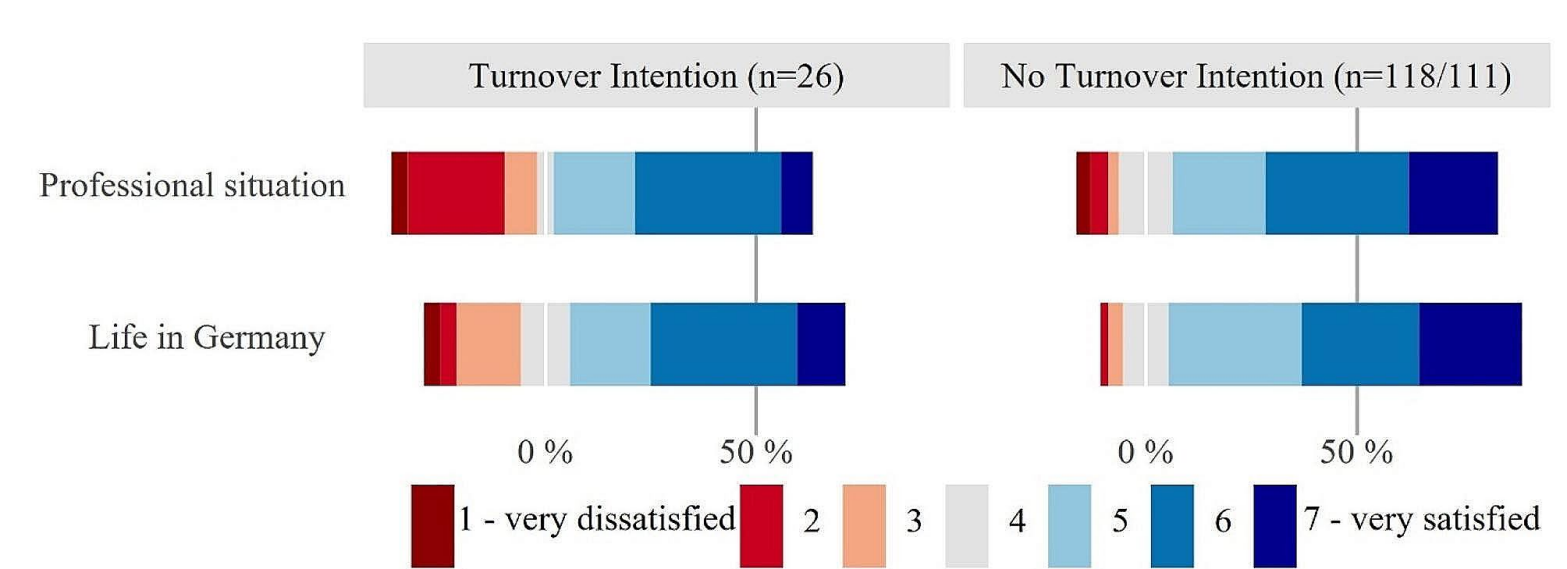
Subgroup comparison of FTP with and without turnover intention on general satisfaction. Legend: Likert plots are centered around the middle category (grey) and stacked to 100%, red color shading represents dissatisfaction, blue indicates satisfaction

**Fig. 4:**
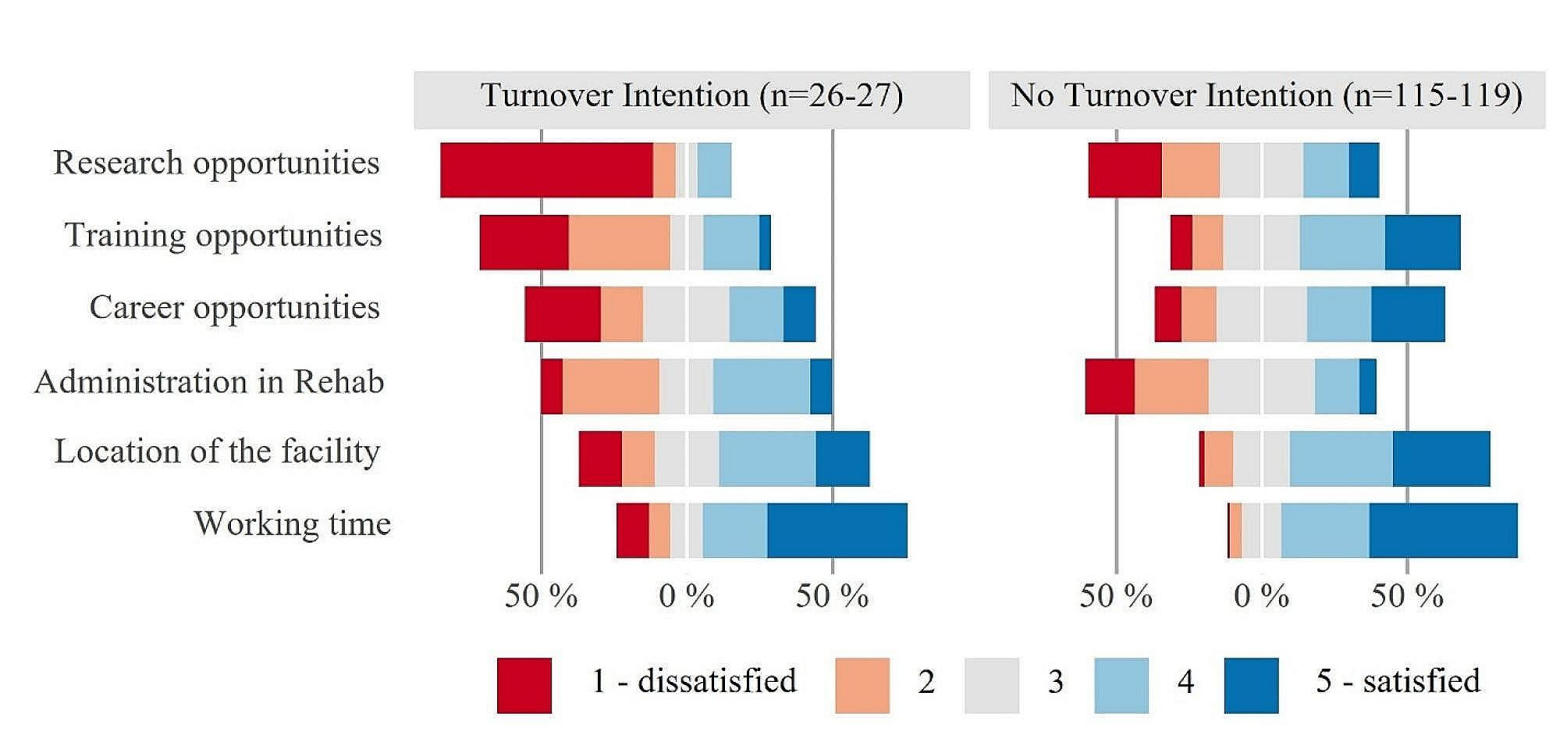
Subgroup comparison of FTP with and without turnover intention on specific aspects of work satisfaction. Legend: Likert plots are centered around the middle category (grey) and stacked to 100%, red color shading represents dissatisfaction, blue indicates satisfaction

**Fig. 5:**
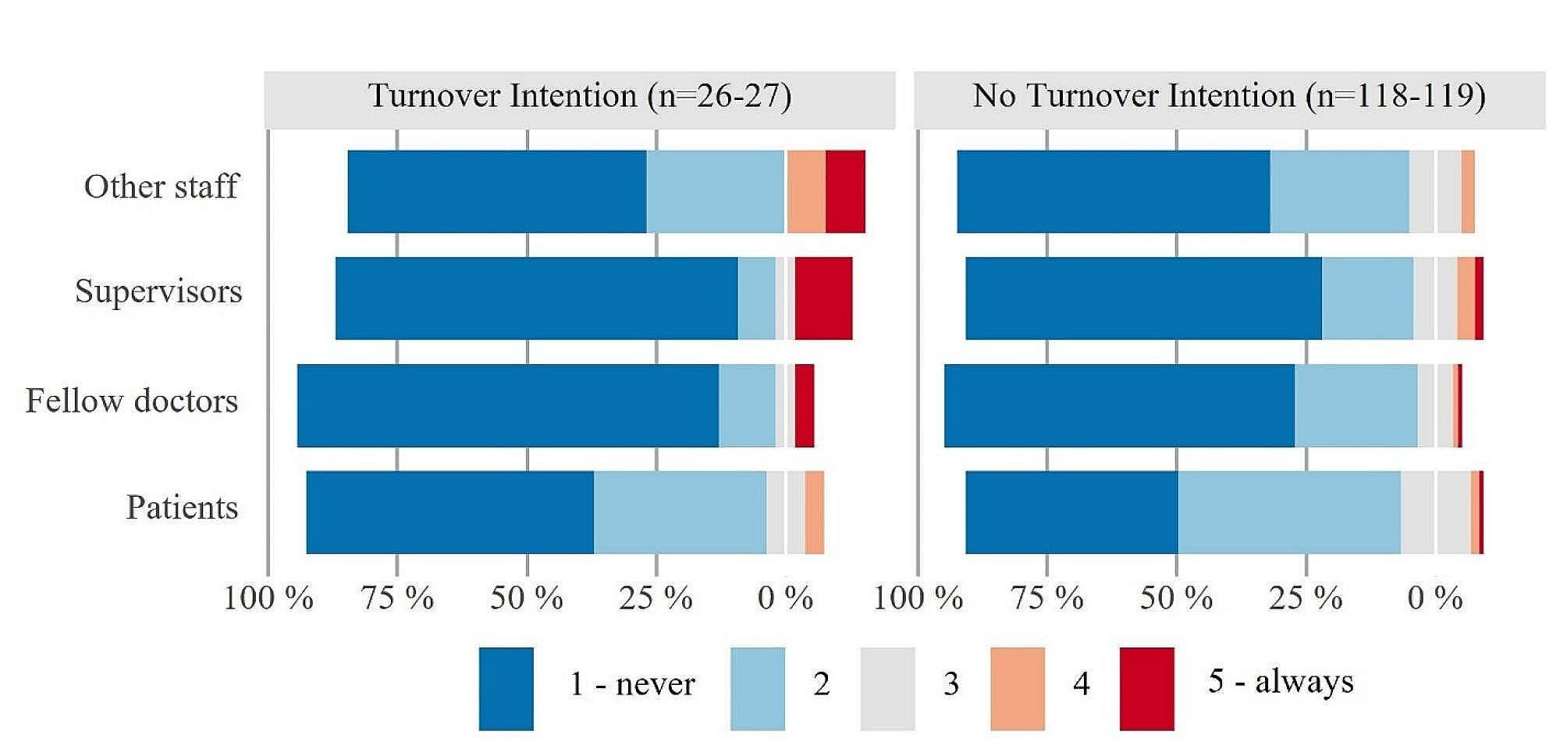
Subgroup comparison of FTP with and without turnover intention on experienced discrimination by different groups. Legend: Likert plots are centered around the middle category (grey) and stacked to 100%, red color shading represents frequent problems with discrimination, blue indicates infrequent problems

Figures 3, 4 and 5 depict general satisfaction as well as specific work-related satisfaction variables and discrimination as Likert plots, centered around the middle category (grey) and stacked to 100% (red color shading represent dissatisfaction/frequent problems with discrimination, blue indicates satisfaction/infrequent problems). Figure 3 shows significant differences in general work-related satisfaction ratings between FTP with and without turnover intention (Fisher-Freeman-Halton exact test 2-tailed *p* = 0.024). Among the FTP with turnover intention (*n* = 26), a total of 34.7% fall into the “dissatisfied” categories, while in the group without turnover intention (*n* = 118), it is only 10.1%. Conversely, 77.1% in the latter group are somewhat to very satisfied. There is also a significant group difference in overall satisfaction with life in Germany (*p* = 0.049). Here, about 1 in 4 FTP with turnover intention (*n* = 26) falls into the red color zone, indicating dissatisfaction, compared to only 1 in 20 in the other group (*n* = 111).

Figure 4 illustrates specific aspects of job satisfaction that are particularly relevant in the field of rehabilitation, arranged according to the increasing satisfaction of FTP with turnover intention (*n* = 26–27). In this group, career-related variables such as “research opportunities” (p = < 0.001) and “training opportunities” (*p* < 0.001) show high levels of dissatisfaction (80.8% and 65.4%, respectively). Among FTP without turnover intention (*n* = 115–119), these figures are significantly lower, at only 45.2% and 18.5%, respectively. Regarding career advancement opportunities, there is also slightly more dissatisfaction in the group with turnover intention, especially in the lowest category (25.9% compared to 9.3% of FTP without turnover intention). However, for the item difficulties with bureaucracy in rehabilitation, both groups show similar ratings, with FTP without turnover intention more often falling into the “dissatisfied” or “rather dissatisfied” categories (40.7% compared to 21.2% of FTP without turnover intention). The differences in both items are not significant (*p* > 0.05). Another significant difference can be seen in the rating of satisfaction with the location of the facility, where FTP with turnover intention tend to be less satisfied than FTP without turnover intention (“rather satisfied” and “satisfied” categories: 51.8% and 68.9%, respectively, *p* = 0.047). Both groups show high satisfaction ratings for working time, with only 1 in 20 FTP without turnover intention falling into the red categories, indicating dissatisfaction, and 18.4% FTP with turnover intention (*p* = 0.081).

Figure 5 displays the frequency of experienced discrimination or unequal treatment by different groups of people. Notably, there is a substantial proportion of ratings in the blue categories, suggesting at first glance that discrimination does not seem to be a frequent phenomenon in either group. The most common experience of discrimination among FTP with turnover intention (*n* = 26) in our sample was from non-medical colleagues (15.4% compared to 2.5% of FTP without turnover intention, *p* = 0.019). The other differences (discrimination experienced from supervisors, medical colleagues, or patients) are not significant. It is noteworthy, however, that in the group of FTP with turnover intention, both extreme categories (“never” and “always”) are more heavily represented than in the group without turnover intention (*p* = 0.097).

## Discussion

The subgroup comparison of FTP with and without turnover intention reveals factors associated with retention within the field of rehabilitation by characterizing the two groups based on their predominant differences. Even though the group with turnover intention is underrepresented in our sample, the subgroup comparison reveals significant differences that can be interpreted as empirical trends. More than half of FTP with turnover intention aim to change to an acute care hospital. Different factors indicate that the FTP with turnover intention are at an earlier career stage: they are significantly (*p* < 0.05) younger, have worked in Germany and in the rehabilitative facilities for a shorter duration and without leading roles in contrast to FTP without turnover intention. At the same time, career-related satisfaction variables like research and training opportunities show the highest levels of dissatisfaction among FTP with turnover intention, indicating that this group may perceive their current role in the field of rehabilitation unfavorable to their professional development.

The regional location of employment is another significant factor influencing retention in the field of rehabilitation. The higher concentration of FTP with turnover intention live in peripheral areas, as opposed to central urban locations, and their lack of satisfaction withr the location of the facility could reflect specific challenges faced by FTP in rural areas as well as differences in lifestyle preferences. Even if discrimination was reported remarkably rarely, FTP with turnover intention report that they have experienced discrimination by colleagues who are not physicians significantly more often than FTP without turnover intention.

From a methodological approach, authors of national and international studies on the intention of physicians with a foreign degree, place of birth, or citizenship to stay or change their job mainly used quantitative online questionnaires and occasionally qualitative interviews. As common definitions and standardized questionnaires are still lacking in the field of physician retention, they asked FTP if they planned a change of job [31], to abandon the specific medical specialty [32], a change of country [33], how long they would stay in the country of migration [34], if they would consider “quitting” [35]. While the focus of this project is on turnover intention in the field of rehabilitation and thus should concern less the individual institution as employer, the methodological procedure in general had to go through several filters (turnover intention in general, direction of change). In addition, it also had to take into account the career-related peculiarities of specialist training by excluding those in the turnover intention group who intend to leave the rehabilitative facility for a time due to the requirements of their residency training, but who would like to return in principle.

Because some of the questions in the international studies used established questionnaires but adapted the surveys specifically to this context, the responses are not directly comparable. For example, in one study [32], two groups were formed (one that intends to stay in the country and one that intends to leave the country) and their independent variables were compared. In another study, the authors formed three groups through class analysis [34] and then compared other characteristics such as age, gender, and motivation. In the present study, we divided participants into two groups, one with an intention to stay and one with a turnover intention. Comparisons with international literature are therefore only possible based on the reasons for turnover intention or to stay.

According to the national and international studies, the following five factors, in descending order of frequency of mention, have a major influence on the intention of migrated physicians or physicians with foreign degrees to leave their position: first, the quality of the working relationship, especially at the management level, is an important determinant of intention to stay. Low turnover intention was achieved by low levels of hierarchy [36], supervision and supportive measures [33, 36], capable leadership [35], good team climate [31], and satisfactory relationships with superiors [32].

In our study, the survey of satisfaction with working relationships with superiors, colleagues, and patients, as well as the survey of difficulties in the team inter-hierarchically and inter-professionally, did not reveal any conspicuous differences in the form of group differences. One hint is provided in the analysis of discrimination, even if self-reported this is rare: If FTP with turnover intention feel discriminated against by their superiors and colleagues who are not physicians more often, it can be assumed that working relationships are impaired and the team climate is not optimal.

Second, a high quality of work environment led to a low turnover intention, insofar as it included the following components: high staffing [35], good task coordination [36], and control of the pace of work [35]. On the other hand, the aspects of threats and violence from patients [35] and a high level of discrimination received from patients in the workplace [31] led to a high turnover intention.

The bureaucracy in rehabilitation facilities was one aspect both FTP with and without turnover intention were rather dissatisfied with. Threats and discrimination from patients are not part of the present study results, but emerge in the evaluation of an open question, which we have addressed in another publication [37].

The third aspect addressed in national and international literature is the personal circumstances in the lives of medical professionals. Three studies examined areas of personal life related to migration and concluded that high satisfaction with life in the new place in general [31, 33] leads to low turnover intention. Furthermore, one study states that country of origin is already a predictor of turnover intention or intention to stay [33]. Having a family in the country to which a medical professional migrates further leads to a very high intention to stay [33, 34].

In our study, FTP with turnover intention have lower satisfaction with life in Germany and also with work in the rehabilitation facility. However, in our sample the family situation did not differ between FTP with and without turnover intention, which could be attributed to a potential sampling bias or underrepresentation of the group who wants to leave in our study.

According to international studies, the fourth group of factors influencing the intention to stay are employment related. These include, for example, a disadvantageous employment contract [33], or barriers to accessing jobs [31]. Interestingly, economic incentives do not necessarily increase the willingness to stay in a job [34].

This aspect is particularly consistent with the results of the present study. FTP with turnover intention are more often in a precarious employment relationship: they have a disadvantageous employment contract, have not been working in the specialist department for long, tend to be more dissatisfied with their working hours and do not hold a management position.

Career and training opportunities are the fifth factor influencing intention to stay. Accordingly, a low turnover intention is more likely if the job offers opportunities for career advancement or further training [32–34].

Again, this shows strong agreement with the present results. The group of FTP with turnover intention is particularly dissatisfied with the career-related items. These are training and development opportunities and opportunities for advancement and research.

The location of the facilities and the social contacts of the FTP were not questioned or mentioned in the national and international studies analyzed here. Both factors are specific to rehabilitation in Germany [20, 21] and also play a central role in our study. That medical institutions located in the periphery have more problems recruiting and retaining their staff is well known [38–40] and reinforces the problem of retention of FTP.

The fact that there are departments with a very high proportion of FTP and—if turnover intention is actually put into practice—a very high turnover compared to departments with few FTP is also not reflected in the national and international literature.

Moreover, the weighting of the most common group differences is centered differently in the present study: while in the studies cited here it is the work relationship and the quality of the work environment that are the most important causes of turnover intention, in the present study it is career biographical factors.

Basically, in our study, the intention to stay increases according to the duration of employment in the facility and in rehabilitation. This “being established” is not found in international literature.

Comparatively younger FTP in an earlier career stage who came to Germany and the rehabilitation departments more recently are overrepresented in the group with turnover intention. This is consistent with the analysis of Becker and Teney [34, 41], who identified a group of so-called career-seekers. Their migration was mainly triggered by the quality and content of employment. They move on when they see a better option.

In our study results, it is apparent that there is a group of “established” FTP, i.e. FTP who have worked in rehab for a long time and whose professional expectations have adapted to the location and orientation of the rehabilitation facilities. These are characterized by a low turnover intention. FTP with a high turnover intention, on the other hand, are often in facilities with many FTP and probably at the same time a high turnover and a rather precarious employment relationship. These facilities need to be systematically supported, as they are most likely needed as steppingstones for the FTP further medical career and have to do an above-average amount of integration work.

### Limitations

A limitation of the FTP survey evaluation is the poor access to this group. In retrospect, the recruitment method via flyers does not seem successful enough and the group of FTP is distorted by self-selection. Such self-selection bias implies that the FTP who did respond may not be representative of the broader, even more heterogeneous, FTP population, especially those with turnover intentions. This limits the generalizability of the findings. Due to the low response rate of the FTP survey, especially due to the low number of FTP with turnover intention (*n* = 27), it was not possible to conduct multivariate regression analysis. Nevertheless, the subgroup comparison revealed tendencies that fit international studies. For our study, we assume that especially FTP with turnover intention did not bother to answer our questions and that this group is much larger than reported by the survey. This underrepresentation could lead to a biased understanding of the professional challenges and needs of FTP with turnover intention. In order to give the analysis a narrow framework, this discussion only refers to FTP and not to other health personnel.

## Conclusion

FTP in rehabilitation facilities are a numerically very relevant phenomenon [37]. Because this study only included physicians with foreign degrees and not, for example, physicians born abroad, the diversity aspect in the rehabilitative departments of the German Pension Insurance (DRV) examined is much greater in reality than we have illustrated using the example of physicians with foreign degrees.

Despite the small sample size, the results show trends, characterizing two groups of FTP: On the one hand, there is a group of “established” FTP, i.e. FTP who have been in Germany for a long time and who have adjusted socially and professionally to their new environment. On the other hand, there is a group of rather younger, career oriented FTP with a high turnover intention, who use their activity in the rehabilitation facility to subsequently gain a foothold in the acute hospital. As our analysis revealed, it is career biographical characteristics that are most strongly related to turnover intention in the field of rehabilitation. Further studies should systematically examine this using a different recruitment strategy, as turnover places a tremendous burden on the rehabilitation system. Discrimination is an issue that does not score conspicuously high in the difficulty scales, but still appears in the group comparison to be related to turnover intention. Here, too, it is worthwhile to initiate measures to raise awareness among supervisors and non-medical staff and to conduct further studies. Future research should aim to employ more inclusive and effective recruitment strategies, ensure a larger and more representative sample, and possibly use more sophisticated statistical analysis to gain a comprehensive understanding of the FTP workforce in the rehabilitation sector.

### Electronic supplementary material

Below is the link to the electronic supplementary material.

Supplementary Material 1

## Data Availability

The datasets used and analyzed during the current study are available from the corresponding author upon reasonable request.

## Footnotes

1 Temporary license granted to non-EU physicians which is typically valid for up to 2 years, during which these doctors are expected to fulfill specific requirements necessary for obtaining their full medical licensure (“Approbation”).

## Declarations

### Open access

I confirm that I understand BMC Health Services Research is an open access journal that levies an article processing charge per articles accepted for publication. By submitting my article, I agree to pay this charge in full if my article is accepted for publication.

### Dual publication

The results/data/figures in this manuscript have not been published elsewhere, nor are they under consideration (from you or one of your Contributing Authors) by another publisher.

### Third party material

All of the material is owned by the authors and/or no permissions are required.
