# Supplementary material for: Turnover intention of foreign trained physicians in German rehabilitation facilities—a quantitative study
Source: BMC Health Serv Res. 2024 Mar 29;24:402. doi: 10.1186/s12913-024-10902-7 (PMC10981321; doi:10.1186/s12913-024-10902-7)
Supplement: Supplementary file 1 — Supplementary Material 1 [file 12913_2024_10902_MOESM1_ESM.pdf]

1. Professional Situation

Are you a **physician with a foreign degree** currently working in a German rehabilitation facility?

☐ yes ☐ no

In which **country** did you **graduate in medicine**?

In which **other countries**, apart from the country where you graduated, did you **study**?

Do you hold a **license to practice medicine (Approbation)** in Germany?

☐ yes ☐ not yet, I have a provisional professional license (Berufserlaubnis) according to §10 Abs. 1 BÄO ☐ no

Since which **year**?

How many **years of professional experience** do you have? (please round to whole years)

in Germany

abroad

What is your **position** at the rehabilitation facility where you currently work?

☐ chief physician ☐ senior physician ☐ assistant physician
☐ ward physician ☐ specialist ☐ intern or visiting physician

Do you have a qualification as **specialized doctor** (Facharzt) in Germany?

☐ yes ☐ not yet, but I am in training for it ☐ no

In which field did you accomplish your **specialized training**?

Which **specialist title** do you want to attain?

1. Professional Situation [Fortsetzung]

In which **rehabilitation department (Fachabteilung)** are you currently working?

- ☐ substance use and dependency-related disorders
- ☐ dermatology
- ☐ gastroenterology
- ☐ internist
- ☐ cardiology
- ☐ children and adolescents
- ☐ neurology
- ☐ oncology
- ☐ orthopaedics
- ☐ pneumology
- ☐ psychosomatics
- ☐ rheumatology
- ☐ other

other

How long have you been working at the rehabilitation facility? (month / year)

/

How many other physicians **with foreign degrees** work in your "Fachabteilung"?

How many physicians **with a German degree** work in your "Fachabteilung"?

How many **hours** are you contracted to work and how many hours do you actually work **per week**?
hours per week listed in the contract

actual hours per week

Do you have a **fixed-term employment contract**? ☐ yes ☐ no

2. Personal and Family Situation

Please specify your **gender.**

- ☐ male
- ☐ female
- ☐ diverse

Please specify your **age.**

- ☐ 26-30 years
- ☐ 31-35 years
- ☐ 36-40 years
- ☐ 41-45 years
- ☐ 46-50 years
- ☐ > 50 years

Please specify your **citizenship(s)**:

2. Personal and Family Situation [Fortsetzung]

What **religion** do you consider yourself part of?

- ☐ Christianity
- ☐ Islam
- ☐ Judaism
- ☐ Hinduism
- ☐ Buddhism
- ☐ not religious
- ☐ other

other

[ ]

Since which year are you in **Germany**?

[ ][ ][ ][ ]

Did you have a **job commitment before you moved** to Germany? ☐ yes ☐ no

For what **reasons** did you **migrate to Germany**? (multiple answers possible)

- ☐ **family** in **Germany**
- ☐ **friends/ social network** in Germany
- ☐ reputable **medical system** in Germany
- ☐ poor **working conditions** in the country of origin
- ☐ poor **training opportunities** in the country of origin
- ☐ low **earnings** in the country of origin
- ☐ poor **living conditions** in the country of origin
- ☐ uncertain **political situation** in the country of origin
- ☐ other

Please state other reasons here:

[ ]

What is your current **family situation**? (multiple answers possible)

- ☐ partner **abroad**
- ☐ partner in **Germany**
- ☐ children **abroad**
- ☐ children in **Germany**
- ☐ **without** partner and **without** children

Do you **financially support** parts of your **family abroad**? Think, for example, of parents, children, grandparents or siblings.

- ☐ yes as **regular** support
- ☐ yes as **occasional** support
- ☐ no

3. Intention to stay

We now want to talk about your professional plans.

	yes	probably yes	maybe	probably not	no
Do you **intend to change** your **current job**?	☐	☐	☐	☐	☐

3. Intention to stay [Fortsetzung]

What career change are you most likely to consider?

- ☐ I will have to change jobs due to my **specialist training requirements**.
- ☐ I would like to transfer to a **different rehabilitation facility** in Germany.
- ☐ I would like to transfer to the **outpatient sector** in Germany.
- ☐ I would like to change to an **acute care clinic** in Germany.
- ☐ I would like to return to my **country of origin** and work there professionally.
- ☐ I would like to migrate to a **different country** and work there professionally.

To which country?

[ ]

When are you most likely to want to **make a career change**? (multiple answers possible)

- ☐ as soon as possible (<1 year)
- ☐ medium-term (1-5 years)
- ☐ longer-term (>5 years)
- ☐ when something suitable occurs
- ☐ after receiving the "Approbation"
- ☐ after completion of the specialist training
- ☐ dependent on a private life event

Can you imagine to work in the field of rehabilitation again after completing your specialist training?

- ☐ yes
- ☐ no

4. Language skills

Native language:

- ☐ other
- ☐ German

Please specify your **native language**:

[ ]

German language skills:

- ☐ almost like native language (**C2**)
- ☐ competent use of language (**C1**)
- ☐ good and independent use of language (**B1** and **B2**)
- ☐ basic knowledge (**A1** and **A2**)

What **other languages** do you speak?

[ ]

	never	rarely	occasionally	often	very often
How often do you experience **difficulties** in your everyday work due to a **lack of German language skills**?	☐	☐	☐	☐	☐
How often do you have **advantages** in your everyday work due to your **other language skills**?	☐	☐	☐	☐	☐

5. Region and Characteristics of the Rehabilitation Facility

We would like to **regionally categorize** the **location of the rehabilitation facility** where you work. The information will be deleted after categorization.

To do so, please provide us with the **zip code (Postleitzahl)** and **town** of the facility where you work.
zip code:

town:

☐ the data may be used for categorization ☐ I would prefer **not to provide details**

Instead, please answer the following two questions for **rough regional classification**.

In which **state (Bundesland)** is your facility located?

☐ Baden-Württemberg	☐ Bavaria	☐ Berlin
☐ Brandenburg	☐ Bremen	☐ Hamburg
☐ Hesse	☐ Mecklenburg-West Pomerania	☐ Lower Saxony
☐ North Row Westphalia	☐ Rhineland-Palatinate	☐ Saarland
☐ Saxony-Anhalt	☐ Saxony	☐ Schleswig-Holstein
☐ Thuringia		

In **what kind of place** is your rehabilitation facility located?

☐ in a **village** in **completely rural environment**	☐ in a **village** near a **city**	☐ in a **small town** with up to 30,000 inhabitants
☐ in a **medium-sized city** with up to 100,000 inhabitants	☐ in a **big city** with up to 500,000 inhabitants	☐ in a **big city** with more than 500,000 inhabitants

What **types of care** does your facility provide? ☐ outpatient ☐ inpatient ☐ both

Approximately, how many **individual treatment places** does your **"Fachabteilung"** have?

Approximately, how many **individual treatment places** does the **whole facility** have in which you work?

6. Support and Measures

Did you have **support** through an **agency** prior to your current employment? ☐ yes ☐ no

What did the agency support you with?

6. Support and Measures [Fortsetzung]

Who supported you since being employed at a rehabilitation facility? (multiple answers possible)

- ☐ German colleagues
- ☐ international colleagues
- ☐ superiors
- ☐ Human Resources Officer
- ☐ Integration Officer
- ☐ other persons in the administration of the facility
- ☐ family
- ☐ friends and acquaintances
- ☐ social networks (e.g. Facebook)
- ☐ external consulting offices
- ☐ no support

What measures to support the professional integration of foreign professionals (physician and non-physician) **exist** in your rehabilitation facility/"Fachabteilung"? (multiple answers possible)

- ☐ mentoring programm
- ☐ peer programm (or other networking opportunity)
- ☐ supervision
- ☐ trainings (e.g. intercultural, leadership, awareness)
- ☐ advanced training (e.g. German health care system, social medicine)
- ☐ support with professional matters (e.g. recognition of foreign degrees)
- ☐ support with private matters (e.g. accommodation, tax declaration)
- ☐ language courses
- ☐ E-learning program
- ☐ culturally sensitive food offered in the canteen/ cafeteria
- ☐ other

Please state the **other measures** here:

What measures to support the professional integration of foreign professionals (physician and non-physician) **would you like to see** in your rehabilitation facility/"Fachabteilung"? (multiple answers possible)

- ☐ mentoring program
- ☐ peer programm (or other networking opportunity)
- ☐ supervision
- ☐ trainings (e.g. intercultural, leadership, awareness)
- ☐ advanced training (e.g. German health care system, social medicine)
- ☐ support with professional matters (e.g. recognition of foreign degrees)
- ☐ support with private matters (e.g. accommodation, tax declaration)
- ☐ language course
- ☐ E-learning program
- ☐ culturally sensitive food offered in the canteen/ cafeteria
- ☐ other

Please state the **other measures** here:

7. Job Satisfaction

Overall, how satisfied are you with your **current professional situation**?

	1	2	3	4	5	6	7	
very unsatisfied	☐	☐	☐	☐	☐	☐	☐	very satisfied

How satisfied are you with the **following aspects** of your **current professional situation**?

	unsatisfied	2	3	4	satisfied
**working hours**	☐	☐	☐	☐	☐
**salary**	☐	☐	☐	☐	☐
**career opportunities**	☐	☐	☐	☐	☐
**further and advanced training opportunities**	☐	☐	☐	☐	☐
**kind of tasks** in rehabilitation	☐	☐	☐	☐	☐
**research opportunities**	☐	☐	☐	☐	☐

7. Job Satisfaction [Fortsetzung]

**professional autonomy**	☐	☐	☐	☐	☐
**workload**	☐	☐	☐	☐	☐
**stress level**	☐	☐	☐	☐	☐
**work-life balance**	☐	☐	☐	☐	☐
**appreciation** of work performance	☐	☐	☐	☐	☐
**relationship to superior**	☐	☐	☐	☐	☐
**relationship to colleagues**	☐	☐	☐	☐	☐
**relationship to patients**	☐	☐	☐	☐	☐
your **rehabilitation facility** as an **employer**	☐	☐	☐	☐	☐

8. Advantages and Difficulties

How often do you have **difficulties** with the following **aspects of your professional life**?
I have difficulties...

	never	rarely	occasionally	often	always
due to a lack of **expertise in social medicine and rehabilitation**	☐	☐	☐	☐	☐
with writing **medical letters and expert opinions** (linguistic)	☐	☐	☐	☐	☐
with writing **medical letters and expert opinions** (professional)	☐	☐	☐	☐	☐
with the **bureaucracy** in the rehabilitation	☐	☐	☐	☐	☐
with **communicating with patients**	☐	☐	☐	☐	☐
with the **interdisciplinary collaboration** in the team (with different professions)	☐	☐	☐	☐	☐
with the **hierarchical collaboration** (with superiors/subordinates)	☐	☐	☐	☐	☐
due to **lack of support measures**	☐	☐	☐	☐	☐
with **discrimination**	☐	☐	☐	☐	☐
due to a **lack of peer group** / because there are few other international medical professionals besides me	☐	☐	☐	☐	☐

In your opinion, what is the **advantage of being trained abroad** for your professional work in Germany?

9. Equal Treatment

Since your employment at the rehabilitation facility, **how often have you experienced personal discrimination and/or unequal treatment** by the following groups of people?

	never	rarely	occationally	often	very often
superiors	☐	☐	☐	☐	☐
physician colleagues	☐	☐	☐	☐	☐
non-physician colleagues	☐	☐	☐	☐	☐
patients	☐	☐	☐	☐	☐

9. Equal Treatment [Fortsetzung]

In what ways have you experienced personal discrimination and/or unequal treatment?

10. Satisfaction with living in Germany

		1	2	3	4	5	6	7	
**Overall**, how satisfied are you with **living in Germany**?	very unsatisfied	☐	☐	☐	☐	☐	☐	☐	very satisfied

How satisfied are you with the following **aspects of life in Germany**?

	unsatisfied	2	3	4	satisfied
**housing situation**	☐	☐	☐	☐	☐
**finances**	☐	☐	☐	☐	☐
**social contacts** in Germany	☐	☐	☐	☐	☐
**location of the facility**	☐	☐	☐	☐	☐
**mobility/transport**	☐	☐	☐	☐	☐
**child care/ school** and **child's education**	☐	☐	☐	☐	☐
**job opportunities** for your **partner**	☐	☐	☐	☐	☐
opportunities for the **acquisition of language skills**	☐	☐	☐	☐	☐
**public administration/bureaucracy**	☐	☐	☐	☐	☐

Closure:

Is your employer facility a **DRV-contracted facility** - i.e., are individual treatment places occupied by DRV? (DRV= Deutsche Rentenversicherung)

☐ yes ☐ no ☐ no idea

Do you have anything else you would like to share with us? Here you have the opportunity.
